# Learning Curve Effect in Reducing Local Recurrence Rate After Resection of Pancreatic Cancer With Arterial Abutment: A Single‐Center Retrospective Study

**DOI:** 10.1002/ags3.70100

**Published:** 2025-09-30

**Authors:** Jun Ishida, Hirochika Toyama, Takayuki Kodama, Yoshihide Nanno, Takuya Mizumoto, Shohei Komatsu, Hiroaki Yanagimoto, Masahiro Kido, Tomoo Ito, Takumi Fukumoto

**Affiliations:** ^1^ Division of Hepato‐Biliary‐Pancreatic Surgery, Department of Surgery Kobe University, Graduate School of Medicine Kobe Japan; ^2^ Department of Diagnostic Pathology Kobe University Graduate School of Medicine Kobe Japan

**Keywords:** borderline resectable, learning curve, local recurrence, locally advanced, pancreatic ductal adenocarcinoma

## Abstract

**Background:**

Pancreatectomy for pancreatic ductal adenocarcinoma (PDAC) with arterial abutment is a complex, high‐risk surgery. We aimed to investigate the clinicopathological features, risk factors, and learning curve for local recurrence after pancreatectomy for PDAC with arterial abutment.

**Methods:**

Sixty consecutive patients who underwent pancreatectomy for borderline resectable and locally advanced PDAC with arterial abutment at Kobe University Hospital between 2010 and 2020 were enrolled in this retrospective study. Logistic regression analysis was performed to investigate the risk factors for local recurrence. The local recurrence rate was examined for every 10 cases over time.

**Results:**

Eighteen (30.0%) patients developed local recurrence; 12 (20.0%) developed local‐only recurrence, and six (10.0%) developed local and other recurrences. The median survival time after surgery was similar between patients with local‐only (*n* = 12) and other recurrences (*n* = 31) (18.9 vs. 17.3 months, *p* = 0.650). The local recurrence rate was significantly lower during the late period than during the early period (13.3% vs. 46.7%, respectively). In the multivariate analyses, the operative period (odds ratio, 7.01; 95% CI: 1.09–45.2; *p* = 0.041) was the independent factor associated with local recurrence. The local recurrence rate in the first 10 patients was 70%, but it gradually decreased to 10% in the last 10 patients.

**Conclusions:**

Local recurrence has a significant impact on survival, as do other recurrences after pancreatectomy for PDAC with arterial abutment. A learning curve may exist for the local recurrence rate, suggesting that it should be performed by experienced surgical teams at high‐volume centers.

## Abstract 2

In a retrospective series of 60 consecutive resections for borderline‐resectable/locally advanced PDAC with arterial abutment, local recurrence rate decreased from 70% in the first 10 cases to 10% in the most recent 10. A learning curve may exist for the local recurrence rate after such surgery, suggesting that it should be performed at high‐volume centers.

## 1 Introduction

Pancreatic ductal adenocarcinoma (PDAC) is an aggressive disease with a 5‐year survival rate of only 13% [1]. It is considered a systemic disease, as it is commonly diagnosed along with distant metastasis [2], and distant recurrence often occurs even after curative resection [3]. However, local recurrence remains significant because a non‐negligible number of patients develop local recurrence without distant metastasis, and their prognosis is as poor as that of patients with distant metastasis [4, 5].

PDAC commonly presents with local progression and invasion of the peripancreatic and periarterial nerve plexuses [6]. As major arteries such as the superior mesenteric artery (SMA), celiac axis (CA), and hepatic artery (HA) are close to the pancreas, PDAC commonly abuts these arteries. Guidelines from the National Comprehensive Cancer Network (NCCN) classify localized PDAC into three categories based on the association between the tumors and major arteries: resectable, borderline resectable (BR), and locally advanced (LA) PDAC [7].

Pancreatectomy for BR/LA PDAC with arterial abutment is technically challenging. Recent studies have demonstrated that 20%–30% of patients develop local‐only recurrence after resection for BR/LA PDAC [8, 9]. Several surgical techniques have been used to prevent local recurrence after pancreatectomy. Arterial divestment [10, 11, 12] and arterial resection [13, 14, 15, 16] are the surgical techniques to perform curative resection for BR/LA PDAC with arterial abutment. In these methods, precise dissection is critically important. Expert surgeons for PDAC pay a careful attention to dissection plane, as an error in dissection plane can lead to residual tumor.

Given the background of PDAC with arterial abutment, the surgeon's experience may play an important role in preventing local recurrence after pancreatectomy. We aimed to investigate the clinicopathological features, risk factors, and learning curve for local recurrence after pancreatectomy for PDAC with arterial abutment.

## 2 Methods

### 2.1 Study Design

We reviewed the data of consecutive patients who underwent pancreatectomy for BR/LA PDAC with major arterial (SMA, CA, or CHA) abutments at Kobe University Hospital between 2010 and 2020. This single‐center retrospective study was approved by the institutional review board in July 2023 (approval number: B230049). Written informed consent was obtained from all patients before initiation of the study. Clinicopathological, operative, and prognostic data of the patients were collected. Factors associated with local recurrence after surgery were also investigated.

### 2.2 Preoperative Evaluation and Treatment

All patients underwent dynamic contrast‐enhanced multidetector computed tomography (MDCT) before treatment initiation. Their resectability status (resectable, BR, LA, and metastatic) was evaluated according to the NCCN guidelines in a multidisciplinary cancer board consisting of experts in hepatobiliary‐pancreatic surgery, gastroenterology, radiology, and oncology. Preoperative treatments, including chemotherapy and chemoradiation, were administered according to the recommendations of the cancer board. In principle, patients with LA PDAC did not undergo upfront surgery. Surgical resection was considered for patients demonstrating substantial tumor shrinkage or maintaining stable disease for a period exceeding 6 months. The treatment strategy for BR PDAC changed during the study period: during the early period, upfront surgery was allowed for BR PDAC, but during the late period preoperative chemotherapy was administered. Two courses of preoperative chemotherapy were adopted as the standard protocol for BR PDAC, while additional courses were administered to some patients depending on their therapeutic response. Chemoradiotherapy was administered to mainly for BR PDAC patients who met the criteria of multi‐institutional studies (e.g., the JASPAC05 trial [17]) and provided informed consent. For patients who had received chemotherapy or chemoradiotherapy at another institution prior to referral to our hospital, the treatment strategy was evaluated by the cancer board at our institution. Preoperative CA 19‐9 was measured immediately before surgery in all patients, but no threshold was set for operative indication.

### 2.3 Surgical Procedures

Standard procedures included pancreaticoduodenectomy, distal pancreatectomy, and total pancreatectomy with D2 lymphadenectomy. All patients underwent surgery performed by board‐certified hepatobiliary‐pancreatic surgeons [18]. Venous resection and reconstruction were performed in patients with suspected portal vein or superior mesenteric vein invasion. In principle, the major arteries were preserved if the tumor was judged to have invaded only up to the nerve plexus. DP‐CAR was performed when tumor invasion of the tunica adventitia at the origin of the splenic artery and CHA or the CA was suspected. HA resection was performed when tumor invasion of the tunica adventitia at the origin of the gastroduodenal artery or HA was suspected. If invasion of the tunica adventitia of the SMA was suspected, resection was aborted. Frozen‐section analyses were performed to distinguish between tumor invasion and fibrosis when tumor invasion was suspected in the dissection plane.

### 2.4 Postoperative Treatment and Surveillance

Postoperatively, adjuvant chemotherapy with S1 or gemcitabine was administered unless the patients refused or were unable to receive it owing to poor performance status. Contrast‐enhanced MDCT was performed, and serum tumor marker levels were measured every 3 months after surgery for postoperative surveillance. Positron emission tomography was performed when recurrence was suspected. Local or lymph node recurrence was suspected when soft tissue was observed along the pancreatic bed or major vessels on MDCT. They were distinguished based on the morphological pattern: infiltrative lesions were diagnosed as local recurrence, and nodular lesions as lymph node recurrence [19].

### 2.5 Statistical Analysis

Continuous variables were expressed as medians with ranges, and categorical variables as percentages. Logistic regression analysis was performed for the univariate and multivariate analyses of parameters potentially associated with local recurrence. Kaplan–Meier analysis and the log‐rank test were performed to estimate the survival outcomes and cumulative local recurrence after surgery. In the Kaplan–Meier analysis for cumulative local recurrence, patients with recurrence other than local recurrence and death from other causes were censored. Statistical significance was set at *p* < 0.05. All analyses were performed using JMP version 14.0.0 (SAS Institute, Cary, NC, USA).

## 3 Results

### 3.1 Patient Characteristics

During the study period, 340 Asian patients underwent pancreatectomies for PDAC. After the exclusion of 255 patients with resectable PDAC, 23 patients with BR PDAC abutting only the portal or superior mesenteric vein, and 2 patients with initially metastatic disease, 60 patients with BR/LA PDAC with arterial abutment (BR, *n* = 49; LA, *n* = 11) were included.

The patient characteristics are shown in Table 1. The median age was 69 years (range, 50–86 years), and 41 patients (68.3%) were male. The abutted arteries were the SMA, CA, and HA in 42 (70.0%), 13 (21.7%), and 24 (40.0%) patients, respectively. Preoperative chemotherapy was administered to 39 (65.0%) patients, including 14 (13.3%) who received radiation therapy. Venous and arterial resections were performed in 38 (63.3%) and 9 (15.0%) patients, respectively. Pathologically, 27 patients (45.0%) underwent R1 resection. Postoperative recurrence was observed in 43 patients (71.7%). Eighteen patients (30.0%) developed local recurrence.

**TABLE 1:** Patient characteristics.

Variables	*N* = 60 (%)
Age	69 (50–86)
Sex, male	41 (68.3)
Resectability	
Borderline resectable	49 (81.7)
Locally advanced	11 (18.3)
Arterial abutment	
SMA	42 (70.0)
CA	13 (21.7)
HA	24 (40.0)
Preoperative chemotherapy	
Yes	39 (65.0)
Gemcitabine + nab‐paclitaxel	19 (31.7)
Folfirinox	4 (6.7)
S1	11 (18.3)
Other	5 (8.3)
With radiation therapy	14 (23.3)
Operative procedure	
PD	43 (71.7)
DP	13 (21.7)
TP	4 (6.7)
Vascular resection	
PV/SMV	38 (63.3)
Arterial resection	9 (15.0)
HA resection	5 (8.3)
CA resection (DP‐CAR)	4 (6.7)
Morbidity (CD Grade 3 or more)	15 (25.0)
90‐day mortality	1 (1.7)
Pathological tumor size, mm	30 (5–107)
Pathological lymph node involvement	42 (70.0)
R status (1 mm rule)	
R0	33 (55.0)
R1	27 (45.0)
Adjuvant chemotherapy	41 (68.3)
Recurrence	43 (71.7)
Local recurrence	18 (30.0)
Local only	12 (20.0)
Local + systemic	4 (6.7)
Local + lymph node	1 (1.7)
Local + remnant pancreas	1 (1.7)
Systemic recurrence	21 (35.0)
Lymph node recurrence	4 (6.7)

Abbreviations: CA, celiac artery; CAR, celiac axis resection; CD, Clavien–Dindo; DP, distal pancreatectomy; HA, hepatic artery; PD, pancreaticoduodenectomy; PV, portal vein; SMA, superior mesenteric artery; SMV, superior mesenteric vein; TP, total pancreatectomy.

Regarding details on preoperative therapy, the median duration of preoperative therapy for patients with BR and LA PDAC was 2 months (range, 2–13 months) and 6 months (range, 2–16 months), respectively. Three patients (BR PDAC, 2 patients (7.1%); LA PDAC, 1 patient (9.1%)) had second‐line chemotherapy before pancreatectomy. Second‐line chemotherapy consisted of S1 and GnP (BR PDAC), and FOLFIRINOX (LA PDAC).

### 3.2 Prognosis of Patients With and Without Local Recurrence

**FIGURE 1:**
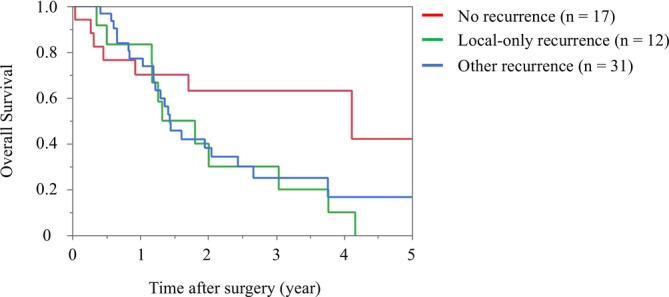
Kaplan–Meier curves of survival after surgery in patients according to recurrence type.

Figure 1 shows the survival curves of patients according to the recurrence type. The median survival time after surgery was similar between patients with local‐only (*n* = 12) and other recurrences (*n* = 31) (18.9 months vs. 17.3 months, *p* = 0.650). The median survival time after surgery was 49.4 months in patients without recurrence (*n* = 17), and there was a significant difference in overall survival between patients with and without recurrence (*p* = 0.043). There were five patients who died within 1 year without recurrence (death from other causes). The causes of death were as follows: postpancreatectomy hemorrhage in two cases, malnutrition in one case, catheter‐related infection in one case, and unknown in one case. Four deaths (from postpancreatectomy hemorrhage, malnutrition, and unknown reason) occurred in the early period (January 2010–March 2018); the other death (from catheter‐related infection) occurred in the late period (April 2018–December 2020).

### 3.3 Factors Associated With Local Recurrence

**FIGURE 2:**
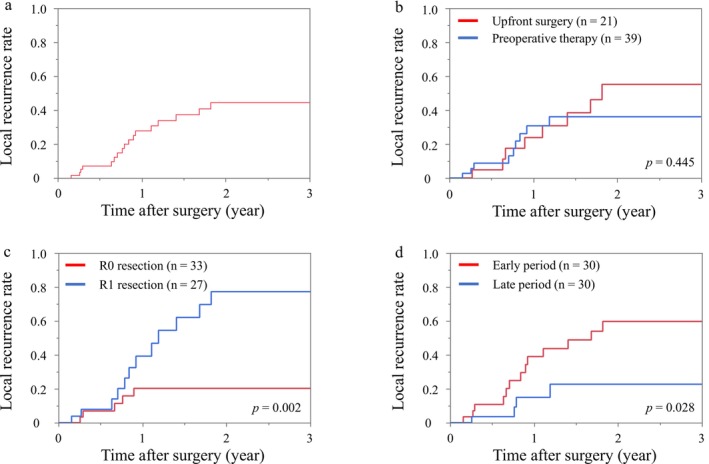
Kaplan–Meier curves of cumulative local recurrence rate. (a) All patients (*n* = 60). (b) Patients with upfront surgery versus those with preoperative chemotherapy. (c) Patients with R0 resection versus those with R1 resection. (d) Patients in the early period versus those in the late period.

**FIGURE 3:**
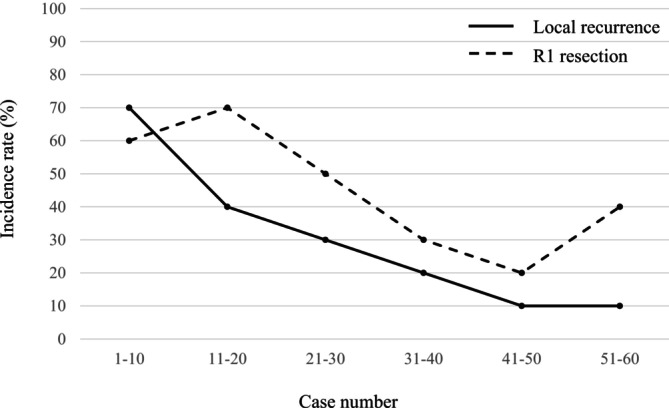
Local recurrence rate and R1 resection rate of 10 cases during the study period.

Table 2 shows the results of the univariate and multivariate analyses for local recurrence. In the univariate analyses, patients who underwent surgery during the early period (January 2010–March 2018) had a higher rate of local recurrence than those who had surgery during the late period (April 2018–December 2020) (46.7% vs. 13.3%, *p* = 0.004). Patients with R1 resection had a higher rate of local recurrence than those with R0 resection (48.2% vs. 15.2%, *p* = 0.005). Pathological tumor size was larger in patients with local recurrence than those without, but the difference was not significant (*p* = 0.090). There was no significant difference in local recurrence rates between BR and LA PDAC among patients who received preoperative therapy for less than 6 months (16.7% vs. 20.0%, *p* = 0.860). However, in patients treated for 6 months or more, the BR group tended to have a higher local recurrence rate compared to the LA group (75.0% vs. 16.7%, *p* = 0.059). In the multivariate analyses, the operative period (odds ratio, 7.01; 95% CI, 1.09–45.2; *p* = 0.041) was the independent factor associated with local recurrence. Figure 2 shows the Kaplan–Meier curves of cumulative local recurrence. In this analysis, we confirmed that the rate of censored patients with recurrence other than local recurrence or death from other causes was comparable across all comparison groups. There was no difference in local recurrence rate between patients with and without preoperative therapy (*p* = 0.445). Patients with R0 resection had lower local recurrence rate than those with R1 resection (*p* = 0.002). Patients in the early period had higher local recurrence rate than those in the late period (*p* = 0.028). Figure 3 shows the changes in the local recurrence rate and R1 resection rate according to the operative period. The local recurrence rate in the first 10 patients was 70%, but it gradually decreased to 10% in the last 10 patients.

**TABLE 2:** Univariate and multivariate multiple regression analyses for local recurrence (*n* = 60).

Variables	Univariate			Multivariate	
	Local recurrence (+), *N* = 18	Local recurrence (−), *N* = 42	*p*	Odds ratio (95% CI)	*p*
Age	66 (51–80)	70 (50–86)	0.313		
Sex					
Male	15 (36.6)	26 (63.4)	0.090	2.49 (0.44–14.0)	0.300
Female	3 (15.8)	16 (84.2)		Ref	
Body mass index	20.5 (16–25.5)	20.4 (17.7–35.2)	0.878		
Resectability					
Borderline resectable	16 (32.7)	33 (67.4)	0.326		
Locally advanced	2 (18.2)	9 (81.8)			
Preoperative CA 19–9^a^	128 (6–19 810)	113 (1–7075)	0.917		
Preoperative therapy					
No	9 (42.9)	12 (57.1)	0.167	2.22 (0.31–15.6)	0.424
Chemotherapy	7 (28.0)	18 (72.0)		6.61 (0.74–58.8)	0.091
Chemoradiation	2 (14.3)	12 (85.7)		Ref	
Duration of preoperative therapy					
< 6 months	5 (17.2)	24 (82.8)	0.156		
≥ 6 months	4 (40.0)	6 (60.0)			
BR, < 6 months preoperative therapy	4 (16.7)	20 (83.3)	0.860		
LA, < 6 months preoperative therapy	1 (20.0)	4 (80.0)			
BR, ≥ 6 months preoperative therapy	3 (75.0)	1 (25.0)	0.059		
LA, ≥ 6 months preoperative therapy	1 (16.7)	5 (83.3)			
Operative procedure					
PD	15 (34.9)	28 (65.1)			
DP	3 (23.1)	10 (76.9)			
TP	0 (0)	4 (100)			
Vascular resection					
PV/SMV	10 (26.3)	28 (73.7)	0.416		
Arterial resection	4 (44.4)	5 (55.6)	0.319		
Operative time, min	537 (442–817)	585 (343–794)	0.341		
Estimated blood loss, mL	485 (120–3616)	735 (130–3215)	0.362		
Pathological tumor size (mm)	35 (20–55)	30 (5–107)	0.090	1.00 (0.97–1.05)	0.828
Pathological lymph node involvement					
Yes	14 (33.3)	28 (66.7)	0.381		
No	4 (22.2)	14 (77.8)			
R status (1 mm rule)					
R1	13 (48.2)	14 (51.9)	**0.005**	4.06 (0.95–17.3)	0.059
R0	5 (15.2)	28 (84.8)		Ref	
Operative period					
Early (January 2010–March 2018)	14 (46.7)	16 (53.3)	**0.004**	7.01 (1.09–45.2)	**0.041**
Late (April 2018–December 2020)	4 (13.3)	26 (86.7)		Ref	

*Note:* Bold values indicate statistical significance (*p* < 0.05).

Abbreviations: DP, distal pancreatectomy; PD, pancreaticoduodenectomy; PV, portal vein; SMV, superior mesenteric vein; TP, total pancreatectomy.

^a^ Measured after the preoperative therapy, if administered.

### 3.4 Comparisons of Operative Characteristics Between the Early and Late Period

Table 3 shows the operative characteristics of patients in the early and late period. There was a significant difference in resectability status between the groups; LA PDAC accounted for 6.7% of cases in the early period and 30.0% in the late period (*p* = 0.016). Preoperative therapy was administered significantly more frequently in the late period than in the early period (86.7% vs. 43.3%, *p* < 0.001). No significant differences were observed in operative time (558 min vs. 585 min, *p* = 0.451), estimated blood loss (695 vs. 725 mL, *p* = 0.888), major morbidity rate (26.7% vs. 23.3%, *p* = 0.766), postpancreatectomy hemorrhage rate (6.7% vs. 6.7%, *p* = 1.000), and 90‐day mortality rate (3.3% vs. 0%) between the groups. The R1 resection rate was significantly higher in the early period compared to the late period (60.0% vs. 30.0%, *p* = 0.019). Although the rate of local recurrence was higher in the early period than in the late period (46.7% vs. 13.3%, *p* = 0.004), the proportion of distant recurrence was lower in the early period than in the late period (33.3% vs. 66.7%, *p* = 0.009). The proportion of patients who died from other causes was higher in the early period than in the late period (20.0% vs. 3.3%, *p* = 0.035).

**TABLE 3:** Comparisons of clinicopathological characteristics between the early and late period.

Variables	Early period	Late period	*p*
	*N* = 30	*N* = 30	
Resectability			
Borderline resectable	28 (93.3)	21 (70.0)	**0.016**
Locally advanced	2 (6.7)	9 (30.0)	
Preoperative therapy	13 (43.3)	26 (86.7)	**< 0.001**
Chemotherapy	5 (16.7)	20 (66.7)	
Chemoradiation	8 (26.7)	6 (20.0)	
Operative procedure			
PD	25 (83.3)	18 (60.0)	
DP	5 (16.7)	8 (26.7)	
TP	0	4 (13.3)	
Vascular resection			
PV/SMV	18 (60.0)	20 (67.7)	0.592
Arterial resection	5 (16.7)	4 (13.3)	0.717
Operative time, min	558 (362–817)	585 (343–794)	0.451
Estimated blood loss, mL	695 (120–3616)	725 (130–1950)	0.888
R status (1 mm rule)			
R1	18 (60.0)	9 (30.0)	**0.019**
R0	12 (40.0)	21 (70.0)	
Major morbidity (CD grade 3a or more)	8 (26.7)	7 (23.3)	0.766
PPH	2 (6.7)	2 (6.7)	1.000
90‐day mortality	1 (3.3)	0	—
Local recurrence	14 (46.7)	4 (13.3)	**0.004**
Local only	10 (33.3)	2 (6.7)	
Local + systemic	4 (13.3)	0	
Local + lymph node	0	1 (3.3)	
Local + remnant pancreas	0	1 (3.3)	
Distant (systemic or lymph node) recurrence	10 (33.3)	20 (66.7)	**0.009**
Distant only	6 (20.0)	19 (63.3)	
Distant + local	4 (13.3)	1 (3.3)	
Death from other causes	6 (20.0)	1 (3.3)	**0.035**

*Note:* Bold values indicate statistical significance (*p* < 0.05).

Abbreviations: DP, distal pancreatectomy; PD, pancreaticoduodenectomy; PPH, postpancreatectomy hemorrhage; PV, portal vein; SMV, superior mesenteric vein; TP, total pancreatectomy.

### 3.5 Local Recurrence Rate According to the Abutted Artery

The local recurrence rates in patients with SMA, CA, and HA abutments were 33.3%, 23.1%, and 12.5%, respectively. Table S1 shows the characteristics of local recurrence in patients with SMA abutments. The extent of abutment on preoperative CT (*p* = 0.789) and tumor abutment of the first jejunal artery (*p* = 0.275) were not associated with the local recurrence rate. The local recurrence rates in patients with abutments to the posterior and right walls of the SMA were 44.4% and 46.2%, respectively, whereas those in patients with abutments to the anterior and left walls of the SMA were 0%. The local recurrence rates in patients with and without arterial divestment of the SMA were 34.5% and 30.8%, respectively.

### 3.6 Factors Associated With Overall Survival

**FIGURE 4:**
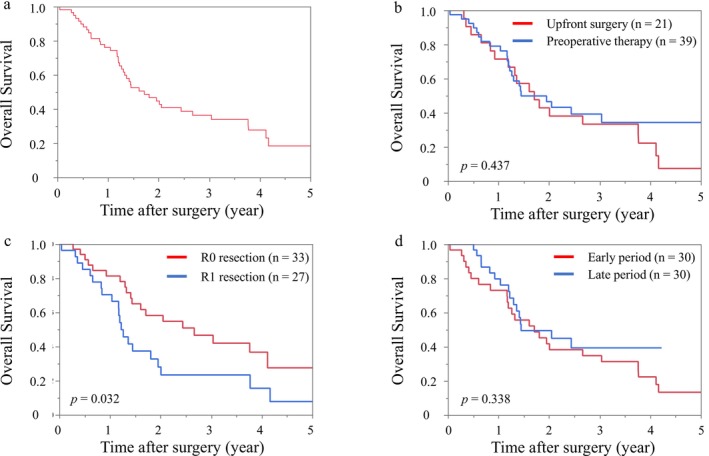
Kaplan–Meier curves of overall survival. (a) All patients (*n* = 60) (b) Patients with upfront surgery versus those with preoperative chemotherapy (c) Patients with R0 resection versus those with R1 resection (d) Patients in the early period versus those in the late period.

Figure 4 shows the Kaplan–Meier curves of overall survival. There was no difference in overall survival between patients with and without preoperative therapy (*p* = 0.437) and those in the early and late period (*p* = 0.338). Patients with R0 resection had better survival than those with R1 resection (*p* = 0.032). In the multivariate Cox regression analysis for overall survival, no variable reached statistical significance. However, higher preoperative CA 19‐9 levels, larger pathological tumor size, and R1 resection showed trends toward association with worse survival (Table 4).

**TABLE 4:** Multivariate Cox regression analysis for overall survival (*n* = 60).

Variables	Multivariate	
	Hazard ratio (95% CI)	*p*
Resectability		
Borderline resectable	0.84 (0.25–3.314)	0.787
Locally advanced	Ref	
Preoperative CA 19–9		
> 37	2.16 (0.90–5.52)	0.084
≤ 37	Ref	
Preoperative therapy		
No	0.36 (0.12–1.07)	0.066
Chemotherapy	0.30 (0.09–1.06)	0.062
Chemoradiation	Ref	
Pathological tumor size (mm)	1.03 (0.99–1.05)	0.051
Pathological lymph node involvement		
Yes	2.00 (0.66–6.62)	0.228
No	Ref	
R status (1 mm rule)		
R1	1.96 (0.91–4.32)	0.085
R0	Ref	
Operative period		
Early (January 2010–March 2018)	1.51 (0.59–4.07)	0.392
Late (April 2018–December 2020)	Ref	

## 4 Discussion

Pancreatectomy for BR/LA PDAC with arterial abutment is complex because it often requires arterial resection or divestment to achieve R0 resection. Recurrence can occur at various sites, including the resection bed, even after a radical resection. The present study showed that local recurrence occurred in 18 (30%) patients, including 12 (20%) patients with local‐only recurrence. The local recurrence rate gradually decreased during the study period, suggesting the existence of a learning curve for local control during pancreatectomy for PDAC with arterial abutment. Patients with local‐only recurrence had a poor prognosis that was similar to that of patients with other recurrences. The prevention of local recurrence is important for better long‐term outcomes after PDAC resection.

The R status is considered a significant factor for long‐term outcomes after PDAC resection. Groot et al. demonstrated that R1 resection (< 1 mm) was an independent risk factor for local‐only recurrence after BR/LA PDAC resection [9]. A recent study analyzing data from two high‐volume centers also demonstrated that R1 resection (< 1 mm) is a predictor of both survival and local recurrence after PDAC resection following neoadjuvant therapy [20]. In our study, R1 resection (< 1 mm) showed a trend toward association with higher local recurrence rate and poorer overall survival, although it did not reach statistical significance in multivariate analysis. Nevertheless, considering previous reports and the nearly significant results observed in our cohort, achieving R0 resection appears to be important for improving prognosis in patients with PDAC with arterial abutment.

In this study, the multivariate analysis showed that the operative period was an independent risk factor for local recurrence. In addition, the gradual decrease in the local recurrence rate during the study period suggested a learning curve for local recurrence after pancreatectomy for PDAC with arterial abutment. Considering previous reports on the learning curve for the local recurrence rate after rectal cancer resection [21, 22], a learning curve could also exist for the local recurrence rate after PDAC resection. Pancreatectomy for PDAC with arterial abutment is challenging, and precise dissection around the major arteries is important to balance oncological and surgical safety during surgery. An inadequate dissection plane can result in residual periarterial tumors and avoidable postoperative complications such as refractory diarrhea or pseudoaneurysm. Dissection of the tumor from the abutting artery is technically demanding but necessary for appropriate judgement during surgery (e.g., in deciding whether the tumor is operable or inoperable, and whether arterial divestment or resection is necessary). As we accumulated experience with cases of R1 resection and local recurrence during the early study period, tumor resections were progressively performed with more meticulous dissection along the vascular structures, similar to the technique employed in the TRIANGLE operation [23]. To ensure safe and precise dissection around major arteries, cold and sharp dissection was increasingly employed at the level of the arterial adventitia. Consequently, the rate of major morbidity including postpancreatectomy hemorrhage did not increase in the late period compared to the early period, despite a higher proportion of patients with LA PDAC. The accumulated surgical experience throughout the study period likely contributed to improved local control and procedural safety in pancreatectomy for PDAC with arterial abutment.

In the current study, five patients died within 1 year without recurrence (death from other causes). Notably, deaths from postpancreatectomy hemorrhage and malnutrition occurred in the early period. Inappropriate dissection planes around major arteries during this period may have contributed to postoperative pseudoaneurysm or excessive resection of the nerve plexus around the SMA. Ensuring an appropriate dissection plane and optimizing perioperative management may help reduce mortality unrelated to recurrence in the early postoperative period.

Preoperative chemotherapy and chemoradiation are used for BR/LA PDAC for the purpose of both treating micro metastasis and local tumor control. Some randomized control trials demonstrated favorable long‐term outcomes after pancreatectomy with preoperative chemotherapy [24, 25]. In the present study, it is possible that changes in treatment strategy during study period affected the results; increased use of preoperative therapy might be the reason for the lower local recurrence rate in the late period. However, as shown in Figure 2b, cumulative local recurrence rate was similar between patients with and without preoperative therapy. A recent report demonstrated similar recurrence site after PDAC resection with and without preoperative chemotherapy [26]. Thus, we consider the lower local recurrence rate in the late period in our study is more likely due to a learning curve in surgical technique than changes in treatment strategy.

Although optimal duration of preoperative therapy remains unclear [27], some studies have reported favorable long‐term outcomes in patients with long‐term preoperative therapy followed by pancreatectomy for PDAC [28, 29]. In contrast, in the present study, patients who received preoperative therapy for 6 months or more tended to have a higher rate of local recurrence compared to those treated for less than 6 months (40.0% vs. 17.2%, *p* = 0.156). In the subgroup analysis according to duration of preoperative therapy, BR PDAC had a higher local recurrence rate than LA PDAC in patients with preoperative therapy for ≥ 6 months (75.0% vs. 16.7%, *p* = 0.059). Because our standard strategy for BR PDAC was surgical resection after two courses of preoperative chemotherapy, a longer duration of preoperative therapy in these patients may reflect a poor treatment response. BR PDAC patients with limited treatment response tend to receive prolonged therapy, which may have contributed to an increased risk of local recurrence.

The local recurrence rate varied based on the abutted artery, the local recurrence rate after resection for PDAC with an SMA abutment was 33.3%, whereas that with an HA abutment was 12.5%. Suzuki et al. reported that a combination of the distance from the SMA and serum CA 19‐9 could predict local recurrence after PDAC resection [30]. A recent study on local recurrence after resection of BR PDAC showed that more than half of the patients with local recurrence developed local recurrence at the SMA [31]. Given our finding that patients with PDAC abutting the SMA from the right and posterior sides had a high rate of local recurrence, it is suggested that an uncinate process lesion abutting the SMA is a high risk for local recurrence. As uncinate process lesions commonly about the jejunal arteries and SMA, arterial divestment and resection of the jejunal arteries are often required. The anatomical feature of jejunal arteries branching from the posterior wall of the SMA makes dissection difficult, possibly increasing the local recurrence around the SMA. Future studies should focus on treatment strategies for PDAC in the uncinate processes abutting the SMA.

This study had several limitations. First, the relatively small sample size may have led to a lack of statistical power. Second, we performed upfront surgery for BR PDAC, especially during the early period, as Japanese guidelines did not recommend preoperative chemotherapy for BR PDAC as a standard treatment during this period. Third, the comparison of local recurrence rates between the early and late periods might be insufficient in terms of the observational time. However, this study was conducted in 2023, more than 2 years after the study period, so we considered the observational time sufficient even for late cases because most local recurrences occur within 2 years after surgery as shown in Figure 2. Finally, although the operative period was an independent factor for local recurrence, it was not associated with overall survival in the multivariate Cox regression analysis. A possible explanation for this discrepancy is the higher rate of distant recurrence in the late period, which may be attributable to the greater proportion of patients with LA PDAC at higher risk of potential distant metastasis. PDAC is a systemic disease in which the development of distant recurrence has a substantial negative impact on overall survival, overshadowing differences in local recurrence rates between operative periods. The prognostic impact of local recurrence should be investigated in future studies. Local recurrence may have a greater impact on overall survival in the future, once improved perioperative systemic chemotherapy is established for PDAC.

## 5 Conclusions

Local recurrence has a significant effect on survival after pancreatectomy for BR/LA PDAC with arterial abutment. R0 resection is important for the prevention of local recurrence and for long‐term survival. A learning curve may exist for the local recurrence rate after pancreatectomy for PDAC with arterial abutment, suggesting that pancreatectomy should be performed by experienced surgical teams at high‐volume centers where many similar surgeries are performed.

## Author Contributions

**Jun Ishida:** conceptualization, data curation, writing – original draft. **Hirochika Toyama:** writing – review and editing, supervision. **Takayuki Kodama:** investigation. **Yoshihide Nanno:** conceptualization, data curation. **Takuya Mizumoto:** conceptualization, data curation. **Shohei Komatsu:** conceptualization. **Hiroaki Yanagimoto:** conceptualization. **Masahiro Kido:** conceptualization. **Tomoo Ito:** investigation. **Takumi Fukumoto:** supervision.

## Ethics Statement

This study was approved by the institutional review board in July 2023 (approval number: B230049).

## Consent

Written informed consent was obtained from all patients before initiation of the study.

## Conflicts of Interest

The authors declare no conflicts of interest.

## Supporting information


**Table S1:** Patients with SMA abutment (*n* = 42).

## Data Availability

The data that support the findings of this study are available from the corresponding author upon reasonable request.
