# Supplementary material for: Learning Curve Effect in Reducing Local Recurrence Rate After Resection of Pancreatic Cancer With Arterial Abutment: A Single‐Center Retrospective Study
Source: Ann Gastroenterol Surg. 2025 Sep 30;10(2):559–69. doi: 10.1002/ags3.70100 (PMC12962044; doi:10.1002/ags3.70100)
Supplement: Supplementary file 1 — Table S1: Patients with SMA abutment (n = 42). [file AGS3-10-559-s001.docx]

| **Table S1. Patients with SMA abutment (n = 42)** | | |  |
| --- | --- | --- | --- |
| Variables | Number of patients | Local recurrence (%) | P value |
| Extent of abutment (preoperative CT) |  |  | 0.789 |
| < 90° | 18 | 7 (38.9) |  |
| 90°- 180° | 20 | 6 (30.0) |  |
| > 180° | 4 | 1 (25.0) |  |
| Tumor abutment of J1A |  |  | 0.275 |
| No | 20 | 5 (25.0) |  |
| Yes | 22 | 9 (40.9) |  |
| Location of tumor abutment of SMA |  |  | - |
| Anterior wall | 9 | 0 |  |
| Left wall | 2 | 0 |  |
| Posterior wall | 18 | 8 (44.4) |  |
| Right wall | 13 | 6 (46.2) |  |
| Arterial divestment |  |  | 0.813 |
| Yes | 29 | 10 (34.5) |  |
| No (preservation of SMA nerve plexus) | 13 | 4 (30.8) |  |

SMA, superior mesenteric artery; J1A, first jejunal artery
